# Data on subgroup specific baseline characteristics and serum sphingosine-1-phosphate concentrations in the Study of Health in Pomerania

**DOI:** 10.1016/j.dib.2017.03.019

**Published:** 2017-03-11

**Authors:** Eileen Moritz, Danilo Wegner, Stefan Groß, Martin Bahls, Marcus Dörr, Stephan B. Felix, Till Ittermann, Stefan Oswald, Matthias Nauck, Nele Friedrich, Rainer H. Böger, Günter Daum, Edzard Schwedhelm, Bernhard H. Rauch

**Affiliations:** a Institute of Pharmacology, Department of General Pharmacology, University Medicine Greifswald, Germany; b Institute of Clinical Pharmacology and Toxicology, University Medical Center Hamburg-Eppendorf, Germany; c German Center for Cardiovascular Research (DZHK), Partner Site Hamburg/Kiel/Lübeck, Germany; d Institute of Pharmacology, Department of Clinical Pharmacology, University Medicine Greifswald, Germany; e Department of Internal Medicine B, University Medicine Greifswald, Germany; f German Center for Cardiovascular Research (DZHK), Partner Site Greifswald, Germany; g Institute for Community Medicine, University Medicine Greifswald, Germany; h Institute of Clinical Chemistry and Laboratory Medicine, University Medicine Greifswald, Germany; i Clinic and Polyclinic for Vascular Medicine, University Heart Center, University Medical Center Hamburg-Eppendorf, Germany

**Keywords:** Clinical and laboratory data, Sphingosine-1-phosphate, Study of Health in Pomerania, Subgroup formation

## Abstract

In this data article, we provide subgroup specific baseline characteristics and serum sphingosine-1-phosphate (S1P) concentrations for healthy individuals within the Study of Health in Pomerania (SHIP)-TREND cohort. After exclusion of subjects with cardiovascular disease, diabetes mellitus, hypertension, metabolic syndrome, elevated liver enzymes and/or chronic kidney disease stadium III or IV, four subgroups were defined according to different limits for body mass index (BMI), alterations in blood lipid levels and smoking status. Tables show respective clinical and laboratory parameters stratified by gender. Serum S1P concentrations are also stratified by age groups. The data presented herein is related to the research article entitled “Reference intervals for serum sphingosine-1-phosphate in the population-based Study of Health in Pomerania” (E. Moritz, D. Wegner, S. Groß, M. Bahls, M. Dörr, S.B. Felix, T. Ittermann, S. Oswald, M. Nauck, N. Friedrich, R.H. Böger, G. Daum, E. Schwedhelm, B.H. Rauch, Clin Chim Acta. 468 (2017) 25–31) [1].

**Specifications Table**

**Table:** 

Subject area	*Medicine*
More specific subject area	*Clinical chemistry, Biomarker development, Sphingosine-1-phosphate*
Type of data	*Tables*
How data was acquired	*Blood pressure: HEM-705CP (Omron, Tokyo, Japan)*
	*HbA*_*1c*_*: Diamat Analyzer (Bio-Rad, Munich, Germany)*
	*Total cholesterol, HDL cholesterol, LDL cholesterol, triglycerides and creatinine: Dimension Vista 500 analytical system (Siemens AG, Erlangen, Germany)*
	*Sphingosine-1-phosphate: liquid chromatography-tandem mass spectrometry; Varian L1200 MS/MS (Agilent Technologies, Waldbronn, Germany)*
Data format	*Analyzed*
Experimental factors	*Blood samples were drawn from the cubital vein of 4.420 participants of the Study of Health in Pomerania (SHIP)-TREND cohort. Aliquots were analyzed immediately or stored at* −80 °C.
Experimental features	*After the definition of different subgroups within the SHIP-TREND cohort, the subgroups were characterized by clinical as well as laboratory parameters and sphingosine-1-phosphate concentrations.*
Data source location	*Greifswald, Germany*
Data accessibility	*Data is with this article.*

**Value of the data**

• The data presented in this DIB article provides further insight into subgroup formation and S1P concentrations depending on BMI limit, altered blood lipid levels and smoking status.
• The data will facilitate the determination of S1P reference intervals in future population-based cohorts.
• The data can be used for establishing S1P as cardiovascular biomarker.

## 1 Data

In this Data in Brief article, we provide clinical as well as laboratory baseline characteristics and serum sphingosine-1-phosphate (S1P) concentrations that extend the results reported in [1] for four different subgroups of healthy individuals within the Study of Health in Pomerania (SHIP)-TREND cohort. In any case, subjects with cardiovascular disease, diabetes mellitus, hypertension, metabolic syndrome, elevated liver enzymes and/or chronic kidney disease stadium III or IV were excluded. With respect to body mass index (BMI), the exclusion limit for the first subgroup was 25 kg/m^2^ (‘BMI<=25’, *n*=805; Table 1) and 35 kg/m^2^ for the second subgroup (‘BMI<=35’, *n*=1474; Table 2), respectively. For the third subgroup altered blood lipid levels were an additional exclusion criterion (‘Normolipidemia’, *n*=740; Table 3) and smoking for the fourth subgroup (‘Non-Smokers’, *n*=884; Table 4), respectively. Here, in each case the BMI limit was defined as 30 kg/m^2^. All parameters were stratified by gender, median serum S1P concentrations also by age groups.

## 2 Experimental design, materials and methods

### 2.1 Study population

The population-based SHIP-TREND cohort comprises 4420 inhabitants of West Pomerania. A detailed description of the cohort study can be found in Völzke et al. [2], [3]. The study conformed to the principles of the Declaration of Helsinki and was approved by the Ethics Committee of the University of Greifswald. All participants provided informed written consent. The exclusion criteria for subgroup formation have been precisely defined in [1].

### 2.2 Clinical and laboratory parameters

Clinical data acquisition and analytical procedures, i.e. for determining laboratory data and S1P concentrations, have been reported previously [1].

### 2.3 Data analysis

Continuous data are given as median (25th and 75th percentiles). The non-parametric Mann–Whitney U-test was used for comparison between women and men.

## Figures and Tables

**Table 1:** Baseline characteristics and serum S1P concentrations of the subgroup ‘BMI<=25’.

	**Women**	**Men**	***p*-Value**
*n*	579	226	
Age, years	39 (31, 48)	37 (27, 46)	0.001
Body mass index, kg/m²	22 (21, 24)	23 (22, 24)	<0.001
Systolic blood pressure, mmHg	109 (102, 118)	121 (114, 129)	<0.001
Diastolic blood pressure, mmHg	70 (65, 75)	73 (68, 79)	<0.001
HbA_1c_, %	4.9 (4.6, 5.3)	5.1 (4.8, 5.4)	<0.001
Total cholesterol, mmol/L	5.10 (4.50, 5.90)	4.75 (4.20, 5.70)	0.001
HDL cholesterol, mmol/L	1.71 (1.47, 1.92)	1.34 (1.14, 1.56)	<0.001
LDL cholesterol, mmol/L	2.94 (2.40, 3.56)	2.92 (2.43, 3.62)	0.512
Triglycerides, mmol/L	0.93 (0.70, 1.25)	1.05 (0.73, 1.43)	0.007
Serum creatinine, µmol/L	66 (59, 73)	80 (73, 89)	<0.001
eGFR, mL/min/1.73 m²	99 (88, 111)	104 (94, 113)	<0.001
Sphingosine-1-phosphate, µM	0.802 (0.687, 0.908)	0.788 (0.688, 0.900)	0.874
20–29 years	0.820 (0.689, 0.921)	0.796 (0.704, 0.912)	
30–39 years	0.806 (0.696, 0.898)	0.796 (0.687, 0.906)	
40–49 years	0.786 (0.679, 0.903)	0.754 (0.669, 0.881)	
50–59 years	0.811 (0.712, 0.895)	0.773 (0.675, 0.892)	
>=60 years	0.735 (0.653, 0.973)	0.871 (0.716, 0.997)	

Continuous data are given as median (25th, 75th percentile), Mann-Whitney U-test was used for comparison between women and men. eGFR, estimated glomerular filtration rate; HbA1c, hemoglobin A1c; HDL, high-density lipoprotein; LDL, low-density lipoprotein.

**Table 2:** Baseline characteristics and serum S1P concentrations of the subgroup ‘BMI<=35’.

	**Women**	**Men**	***p*-Value**
*n*	969	505	
Age, years	41 (33, 51)	40 (30, 51)	0.058
Body mass index, kg/m²	24 (22, 27)	25 (23, 27)	<0.001
Systolic blood pressure, mmHg	111 (104, 119)	124 (116, 130)	<0.001
Diastolic blood pressure, mmHg	71 (67, 76)	75 (70, 80)	<0.001
HbA_1c_, %	5.0 (4.7, 5.3)	5.1 (4.8, 5.4)	<0.001
Total cholesterol, mmol/L	5.20 (4.60, 6.00)	5.10 (4.40, 5.80)	0.003
HDL cholesterol, mmol/L	1.66 (1.44, 1.89)	1.30 (1.13, 1.52)	<0.001
LDL cholesterol, mmol/L	3.09 (2.48, 3.68)	3.22 (2.60, 3.83)	0.010
Triglycerides, mmol/L	0.98 (0.74, 1.32)	1.16 (0.82, 1.60)	<0.001
Serum creatinine, µmol/L	67 (59, 73)	83 (75, 91)	<0.001
eGFR, mL/min/1.73 m²	98 (86, 110)	99 (89, 111)	0.014
Sphingosine-1-phosphate, µM	0.808 (0.694, 0.920)	0.797 (0.701, 0.922)	0.653
20–29 years	0.820 (0.690, 0.931)	0.781 (0.703, 0.919)	
30–39 years	0.807 (0.689, 0.912)	0.813 (0.697, 0.939)	
40–49 years	0.806 (0.694, 0.925)	0.782 (0.703, 0.910)	
50–59 years	0.805 (0.705, 0.904)	0.807 (0.702, 0.942)	
60–69 years	0.851 (0.694, 0.999)	0.818 (0.718, 0.928)	
>=70 years	0.762 (0.674, 0.913)	0.721 (0.619, 0.872)	

Continuous data are given as median (25^th^, 75th percentile), Mann–Whitney U-test was used for comparison between women and men. eGFR, estimated glomerular filtration rate; HbA1c, hemoglobin A1c; HDL, high-density lipoprotein; LDL, low-density lipoprotein.

**Table 3:** Baseline characteristics and serum S1P concentrations of the subgroup ‘Normolipidemia’.

	**Women**	**Men**	***p*-Value**
*n*	524	216	
Age, years	38 (31, 45)	34 (27, 46)	0.033
Body mass index, kg/m²	23 (21, 26)	25 (22, 26)	<0.001
Systolic blood pressure, mmHg	110 (103, 118)	123 (115, 130)	<0.001
Diastolic blood pressure, mmHg	70 (66, 75)	73 (68, 79)	<0.001
HbA_1c_, %	4.9 (4.6, 5.2)	5.0 (4.8, 5.3)	<0.001
Total cholesterol, mmol/L	4.70 (4.20, 5.20)	4.40 (4.10, 4.90)	<0.001
HDL cholesterol, mmol/L	1.67 (1.46, 1.90)	1.39 (1.18, 1.56)	<0.001
LDL cholesterol, mmol/L	2.65 (2.23, 2.97)	2.63 (2.26, 3.02)	0.221
Triglycerides, mmol/L	0.89 (0.66, 1.16)	0.90 (0.64, 1.23)	0.639
Serum creatinine, µmol/L	67 (59, 73)	83 (75, 91)	<0.001
eGFR, mL/min/1.73 m²	99 (88, 112)	102 (92, 113)	0.114
Sphingosine-1-phosphate, µM	0.805 (0.686, 0.918)	0.788 (0.682, 0.920)	0.531
20–29 years	0.812 (0.678, 0.935)	0.785 (0.705, 0.881)	
30–39 years	0.801 (0.685, 0.902)	0.838 (0.680, 0.954)	
40–49 years	0.807 (0.691, 0.920)	0.754 (0.673, 0.883)	
50–59 years	0.814 (0.701, 0.885)	0.827 (0.703, 0.973)	
60–69 years	0.870 (0.644, 1.069)	0.797 (0.652, 0.893)	
>=70 years	0.745 (0.692, 0.768)	0.612 (0.503, 0.810)	

Continuous data are given as median (25th, 75th percentile), Mann–Whitney U-test was used for comparison between women and men. eGFR, estimated glomerular filtration rate; HbA1c, hemoglobin A1c; HDL, high-density lipoprotein; LDL, low-density lipoprotein.

**Table 4:** Baseline characteristics and serum S1P concentrations of the subgroup ‘Non-Smokers’.

	**Women**	**Men**	***p*-Value**
*n*	590	294	
Age, years	42 (34, 53)	41 (32, 51)	0.064
Body mass index, kg/m²	24 (22, 26)	25 (23, 27)	<0.001
Systolic blood pressure, mmHg	111 (104, 120)	124 (116, 130)	<0.001
Diastolic blood pressure, mmHg	71 (66, 76)	74 (70, 79)	<0.001
HbA_1c_, %	5.0 (4.6, 5.3)	5.1 (4.8, 5.4)	<0.001
Total cholesterol, mmol/L	5.30 (4.60, 6.10)	5.10 (4.40, 5.80)	0.001
HDL cholesterol, mmol/L	1.71 (1.48, 1.97)	1.33 (1.17, 1.52)	<0.001
LDL cholesterol, mmol/L	3.08 (2.47, 3.70)	3.18 (2.61, 3.82)	0.091
Triglycerides, mmol/L	0.94 (0.70, 1.29)	1.09 (0.79, 1.52)	<0.001
Serum creatinine, µmol/L	67 (59, 74)	84 (76, 91)	<0.001
eGFR, mL/min/1.73 m²	97 (85, 108)	98 (88, 109)	0.096
Sphingosine-1-phosphate, µM	0.802 (0.685, 0.910)	0.782 (0.698, 0.908)	0.934
20–29 years	0.828 (0.696, 0.933)	0.771 (0.684, 0.860)	
30–39 years	0.801 (0.682, 0.901)	0.854 (0.703, 0.969)	
40–49 years	0.783 (0.683, 0.896)	0.776 (0.700, 0.904)	
50–59 years	0.808 (0.699, 0.896)	0.795 (0.705, 0.871)	
60–69 years	0.813 (0.668, 1.010)	0.806 (0.684, 0.903)	
>=70 years	0.766 (0.673, 0.916)	0.705 (0.583, 0.944)	

Continuous data are given as median (25th, 75th percentile), Mann–Whitney U-test was used for comparison between women and men. eGFR, estimated glomerular filtration rate; HbA1c, hemoglobin A1c; HDL, high-density lipoprotein; LDL, low-density lipoprotein.
